# Zinc Supplementation and Ischemia Pre-conditioning in Renal Ischemia/Reperfusion Injury

**DOI:** 10.21315/mjms2019.26.4.5

**Published:** 2019-08-29

**Authors:** Bahar Mazaheri, Fatemeh Emami, Fatemeh Moslemi, Ardeshir Talebi, Mehdi Nematbakhsh

**Affiliations:** 1 Water and Electrolytes Research Center, Isfahan University of Medical Sciences, Isfahan, Iran; 2 Department of Clinical Pathology, Isfahan University of Medical Sciences, Isfahan, Iran; 3 Department of Physiology, Isfahan University of Medical Sciences, Isfahan, Iran; 4 Isfahan^MN^ Institute of Basic and Applied Sciences Research, Isfahan, Iran

**Keywords:** renal ischemia/reperfusion, zinc, pre-conditioning ischemia, nephrectomy, rat

## Abstract

**Backgrounds:**

Renal ischemia/reperfusion (RIR) is a major cause of kidney dysfunction in clinic. The main objective of this study was to investigate the effect of pre-conditioning ischemia (IPC) and zinc (Zn) supplementation on renal RIR injury.

**Methods:**

A total of 63 unilateral nephrectomised male and female Wistar rats were divided into five groups. Group 1 (ShOPR): Rats as sham-operated group were subjected to surgical procedure without RIR. Group 2 (Isch): Rats underwent RIR (left kidney ischemia for 30 min followed by 48 h reperfusion). Group 3 (Zn+Isch): Rats were treated as group 2 but they received Zn sulphate (30 mg/kg) 1 h before induction of RIR. Group 4 (IPC+Isch): Rats were treated as group 2 but they underwent 1 min of ischemia followed by 3 min reperfusion as IPC, which was repeated for three times before induction of RIR. Group 5 (Zn+IPC+Isch): Rats were subjected to receive both Zn sulphate and IPC before induction of RIR. Urine samples were collected in the last 6 h of reperfusion, and finally biochemical and histological measurements were performed.

**Results:**

The serum level of creatinine (Cr), normalised kidney weight (KW) and kidney tissue damage score (KTDS) increased by RIR alone significantly (*P* < 0.05). These parameters were attenuated statistically by Zn supplementation (*P* < 0.05). However, IPC alone or co-treatment of Zn and IPC did not improve the biochemical and histological markers altered by RIR injury.

**Conclusion:**

Zn supplementation had a protective role against RIR while such protective effect was not observed by IPC alone or by co-treatment of Zn and IPC.

## Introduction

Renal ischemia/reperfusion (RIR) injury is a complex phenomenon, which disturbs renal function and induces kidney tissue damages (1, 2) resulting in acute kidney injury (AKI) (3). Clinically, RIR injury is occurred in different circumstances such as renal transplantation, renal surgeries, post-resuscitation shock, complex cardiovascular surgeries, and chemical agents/drugs induced renal toxicity (4, 5). Renal transplantation induced RIR injury increases renal dysfunction and mortality rate (3, 5). In general, ischemia followed by reperfusion induces reactive oxygen species (ROS) formation and inflammation, oxidative stress, mitochondrial damage, and apoptosis, which all contribute to renal disorder (3, 4). In addition, it is well known that the rate, intensity, and prognosis of kidney disease and RIR injury are sex-related.

In past years, special attentions have been made to suggest clinical protocols and strategies to limit the RIR-induced kidney dysfunctions. Among these, antioxidants supplementation was more prominent, while pre-conditioning ischemia (IPC) strategy also was considered to protect the kidney against main RIR injury (6). In IPC strategy, the organ will be subjected to brief and sequential time periods ischemia/reperfusion (IR) before the main ischemia will be performed; therefore the increased tissue resistance to IR will be resulted (7). The IPC efficacy depends on time and repeating sequence of IR. Although, the exact mechanism of this method is not fully documented, the reduction in inflammatory response and cellular apoptosis have been suggested as the proposed mechanism (8). The pharmacological IPC is also another interesting method in which chemical compounds will be used to suppress the inflammation and to increase antioxidant effect (9, 10), because during RIR injury, the activity of free radical scavenger enzymes such as superoxide dismutase (SOD), catalase (CAT), and glutathione peroxidase (GPx) play an important role to reduce ROS and tissue inflammation (11). Treatment with cobalt salts such as cobalt chloride (CoCl_2_) has also shown protective effect on RIR injury (12). The optimal duration of IPC is extremely important, and the net definite optimal time has not been defined yet. In the study by Choi and colleagues, 3 min, 5 min and 7 min of ischemia followed by 10 min of reperfusion as IPC was performed (3) while others used 4 min or 5 min of ischemia followed by 11 min or 5 min of reperfusion as IPC (4, 6, 8). However; the shorter the time of IPC, the better would be the practice in the clinic. Therefore, in this study, we evaluated three cycles of 1 min ischemia followed by 3 min reperfusion.

Zinc (Zn) is an essential element for the stability and activity of SOD, which its antioxidant effect was reported in RIR model (13, 14). Subcutaneous injection of Zn chloride also showed an intensive protective effect in reduction of tubular injury and necrosis. This trace element attenuated the increased serum levels of urea and creatinine (Cr) induced by RIR injury (13, 15). Further studies may verify its essential protective role against RIR injury. Therefore, in the current study the effect of single dose of Zn and three cycles of 1 min ischemia followed by 3 min of reperfusion as IPC against RIR injury was evaluated.

## Materials and Methods

### Animals

To determine the sample size, the dependent variable of Cr was considered to compare the mean values between the groups. Based on a previous study (16), the mean difference between sham and RIR groups in serum Cr level was about 0.45 (*d* = 0.45) with standard deviation of 0.2 (σ = 0.2). The number of experimental groups in this study was five, and for two-tailed significance level of 0.05 (α = 0.05, Z1-α/2 = 1.96), 80% test power (β = 0.2, Z1-β = 0.80), and using G*Power software version 3.1.9.4 for one way ANOVA test, the total sample size was calculated as about 65 animals. Accordingly a total of 63 male and female rats were used in this study. These animals were randomly (by statistical random table) divided into five groups. Therefore, the experiment was conducted in 31 male (200 g–277 g) and 32 female (164 g–220 g) Wistar rats.

### Study Design

The animals were anesthetised with chloral hydrate (450 mg/kg, intraperitoneally) (Merck, Germany) and the animals were subjected to right kidney nephrectomy. After one week of recovery, the rats were randomly divided into five experimental groups defined as the followings:

- Group1 (called ShOPR, *n* = 7 males and *n* = 8 females): The rats were subjected to surgical procedure without RIR.
- Group 2 (called Isch, *n* = 6 males and *n* = 7 females): The animals were subjected to RIR surgery by an incision that was made on the abdominal skin, the left kidney was pulled out gently and the vessels were clamped for 30 min followed by 48 h reperfusion.
- Group 3 (called Zn+Isch, *n* = 7 males and *n* = 5 females): The rats received Zn sulphate (30 mg/kg) and after 1 h they were subjected to RIR as described for group 2.
- Group 4 (called IPC+Isch, *n* = 5 males and *n* = 6 females): The animals’ renal vessels were clamped for 1 min followed by 3 min reperfusion, and this action was repeated three times (called IPC), then the animals were subjected to RIR as described for group 2.
- Group 5 (called Zn+IPC+Isch, *n* = 6 males and *n* = 6 females): The rats received Zn sulphate (30 mg/kg) 1 h before they were subjected to IPC and RIR as described for group 4.

### Urine Collection

All the rats were subjected to be kept in standard metabolic cage for urine collection in the last 6 h of reperfusion.

### Blood Samples and Measurements

After 48 h of reperfusion, the animals were anesthetised again, and the blood samples were taken via heart puncture, and the sera were stored in −20 °C. The serum and urine levels of Cr were determined using quantitative diagnostic kits (Pars Azmoon, Iran) and automatic analyzer RA-1000 (Technicon, Ireland). The urine and plasma sodium concentrations were measured by phlamephotomer assay.

The sample of each kidney was fixed in 10% formalin for hematoxylin-eosin (H&E) staining to determine the kidney tissue damage score (KTDS) by a pathologist who was blinded to the study protocol. The serum levels of malondialdehyde (MDA) and nitrite (Greiss Method) were measured. The clearance Cr (ClCr), urine flow rate (UF), and the percentage of excretion Na fraction (ENa fraction) were also determined.

### Statistical Analysis

The data were reported as mean±(SD). One-way ANOVA test was used to analyse the data. Mann-Whitney or Kruskal-Wallis tests were applied to compare the KTDS between the groups.

## Results

The results revealed that the serum level of Cr increased significantly in Isch group compared with ShOPR group (*P* < 0.05). In Zn+Isch group, the serum level of Cr decreased significantly compared with Isch group (*P* = 0.004) (Table 1), and such finding was not seen in IPC+Isch and Zn+IPC+Isch groups. The left kidney weight (KW) showed significant increment in all Isch groups compared with ShOPR group, however this parameter in IPC+Isch+Zn group was increased significantly compared with Isch group (*P* < 0.05) (Table 2). The results of KTDS indicated that kidney injury increased significantly in Isch groups compared with ShOPR group (*P* < 0.05) and Zn supplementation improved kidney injury significantly in Zn+Isch group compared with Isch group (*P* < 0.05) (Table 3).

Although the ClCr was decreased in IPC+Isch group compared with ShOPR group and it was increased in Zn+IPC+Isch group compared with Isch group, these changes were not significant (Table 4). The ENa fraction in Zn+IPC+Isch group decreased insignificantly compared with ShOPR or Isch group (Table 5). No significant difference was found in urine flow (UF) rate between the groups (Table 6). The results related to the serum levels of MDA and nitrite showed no significant differences between the groups (Tables 7 and 8). The kidney tissue images of all the experimental groups are demonstrated in Figure 1. More damages were observed in Isch, IPC and Zn+IPC+Isch groups compared with ShOPR group.

## Discussion

The main purpose of this study was to determine the protective effect of Zn and IPC in RIR injury. The results showed that Zn supplementation protects the kidney against RIR injury, while IPC implementation and co-treatment of Zn and IPC did not show such a protective effect. The RIR injury may activate some pathological pathways such as neutrophils activation, and ROS releasing, and promotes other inflammatory process, which all cause kidney tissue damage (17). Inflammatory mediators lead to disturb the epithelial cells and decrease renal blood flow (1, 18). It is also expected that the RIR injury induced inflammation may increase KW as Afyouni and colleagues demonstrated that KW increased in RIR injury model (19). A model of IPC, in which bilateral renal pedicles were clipped for 5 min followed by 5 min reperfusion for three times, resulted in an improvement of renal injury (20). Such improvement was not obtained in our experiment. This discrepancy between our results and other findings may be related to vessel closure duration to induce IPC. Possibly the 1 min vessel clamp followed by 3 min reperfusion repeated for three times was not completely enough to prepare the kidney for the main RIR since it is reported that 5 min IPC performed the best protective effect against RIR injury (3). Also the kidney cortex blood flow is directly under the influence of renal artery blood flow, but the renal medulla blood flow is slightly different. It is reported that oxygen disappearance rate during complete kidney ischemia is different between renal cortex and medulla (21), and the outer medulla hypoxia may remain during reperfusion after RIR (22). Therefore, we may assume that 1 min closure of the kidney vessels may not be adapted for the whole kidney against major RIR. Accordingly, a longer ischemia time is suggested to have an efficient IPC (3, 20, 23).

Zn as a protective agent against RIR was studied before, and it was shown that Zn improved RIR injury (14, 24, 25). Possibly, the antioxidant effect of Zn (26) is the major cause of this protection since Zn increases metallothionein production, which scavenges free radicals (OH) (27) and Zn deficiency disturbs lipid peroxidation system (28). Barekat and colleagues reported that Zn protected the kidney against RIR in ovariectomised rats (24) while the protective role of Zn against RIR injury reported to be sex-related (29). On the contrary, co-treatment of Zn and IPC did not protect the kidney against RIR. At this time, there is no interpretation for such finding, but the interaction between Zn and IPC may abolish the protective effect of Zn alone as it was demonstrated in Zn+Isch group.

## Conclusion

The model of IPC in this study did not protect the kidney against ischemia. However, a single dose of Zn administration before RIR improves RIR injury. But IPC accompanied by Zn did not protect the kidney against RIR injury with unknown mechanism.

## Figures and Tables

**Table 1:** Comparison of mean serum levels of Cr between the five groups

Groups	*n*	Creatinine (mg/dL) Mean (SD)	*F*-statistic (df1, df2)	*P*-value	Group versus ShOPR	Group versus Isch	Group versus Zn+Isch	Group versus IPC+Isch
ShOPR	15	0.76 (0.11)			-	-	-	-
Isch	13	0.95 (0.18) *	4.52 (4, 58)		0.002	-	-	-
Zn+Isch	12	0.80 (0.14)†		0.004	0.490	0.019	-	-
IPC+Isch	11	0.96 (0.16) *			0.001	0.834	0.014	-
Zn+IPC+Isch	12	0.90 (0.12) *			0.024	0.403	0.127	0.314

One-way ANOVA, post-hoc LSD analysisshowssignificantdifference between groups. The asterisk (*) indicates significant difference in Isch, IPC+Isch, Zn+IPC+Isch from ShOPR group (*P* = 0.002, *P* = 0.001 and *P* = 0.024, respectively) and the symbol (†) indicates from Isch group (*P* = 0.019)

**Table 2:** Comparison of mean KW between the five groups

Groups	*n*	KW(g)/100gBW Mean (SD)	*F*-statistic (df1, df2)	*P*-value	Group versus ShOPR	Group versus Isch	Group versus Zn+Isch	Group versus IPC+Isch
ShOPR	15	0.45 (0.04)			-	-	-	-
Isch	13	0.55 (0.06)*	6.99 (4, 57)		< 0.001	-	-	-
Zn+Isch	12	0.51 (0.05)*		< 0.001	0.002	0.099	-	-
IPC+Isch	11	0.51 (0.05)*			0.004	0.093	0.912	-
Zn+IPC+Isch	12	0.49(0.04)*†			0.042	0.007	0.271	0.347

One-way ANOVA, Post-hoc LSD analysisshowssignificantdifference between groups. The asterisk (*) indicates significant difference in Isch, Zn+Isch, IPC+Isch, Zn+IPC+Isch from ShOPR group (*P* = < 0.001, *P* = 0.002, *P* = 0.004 and *P* = 0.042, respectively) and the symbol (†) indicates from Isch group (*P* = 0.007)

**Table 3:** Comparison of mean KTDS between the five groups

Groups	*n*	KTDS Mean (SD)	Test statistic (df)	*P*-value	Group versus ShOPR	Group versus Isch	Group versus Zn+Isch	Group versus IPC+Isch
ShOPR	15	0.8 (0.41)			-	-	-	-
Isch	12	2.16 (0.38)*	33.83 (4)		< 0.001	-	-	-
Zn+Isch	12	1.41(0.51)*†		< 0.001	0.004	0.002	-	-
IPC+Isch	10	2.10 (0.31)*			0.000	0.658	0.003	-
Zn+IPC+Isch	11	2.00 (0.89)*			0.001	0.630	0.101	0.782

The Mann-Whitney or Kruskal-Wallis analysisshowssignificantdifference between groups. The asterisk (*) indicates significant difference in Isch, Zn+Isch, IPC+Isch and Zn+IPC+Isch from ShOPR group (*P* < 0.001, *P* = 0.004, *P* < 0.001, *P* = 0.001) and the symbol (†) indicates from Isch group (*P* = 0.002)

**Table 4:** Comparison of mean ClCr between the five groups

Groups	*n*	ClCr (μL/min/g tissue) Mean (SD)	*F*-statistic (df1, df2)	*P*-value	Group versus ShOPR	Group versus Isch	Group versus Zn+Isch	Group versus IPC+Isch
ShOPR	15	82.00 (40.28)			-	-	-	-
Isch	13	60.43 (28.93)	1.19 (4, 56)		0.147	-	-	-
Zn+Isch	12	78.85 (41.71)		0.162	0.834	0.232	-	-
IPC+Isch	11	59.05 (45.62)			0.151	0.931	0.230	-
Zn+IPC+Isch	12	83.68 (33.27)			0.911	0.133	0.757	0.136

One-way ANOVA, Post-hoc LSD analysis shows no significant difference between groups

**Table 5:** Comparison of mean excretion Na fraction (ENa fraction) between the five groups

Groups	*n*	ENa Fraction (%) Mean (SD)	*F*-statistic (df1, df2)	*P*-value	Group versus ShOPR	Group versus Isch	Group versus Zn+Isch	Group versus IPC+Isch
ShOPR	15	0.70 (0.35)			-	-	-	-
Isch	13	0.69 (0.45)	0.96 (4, 53)		0.937	-	-	-
Zn+Isch	12	0.63 (0.52)		0.432	0.722	0.784	-	-
IPC+Isch	11	0.78 (0.81)			0.736	0.686	0.514	-
Zn+IPC+Isch	12	0.34(0.46)			0.114	0.136	0.222	0.078

One-way ANOVA, Post-hoc LSD analysis shows no significant difference between

**Table 6:** Comparison of mean UF between the five groups

Groups	*n*	UF (μL/min/g tissue) Mean (SD)	*F*-statistic (df1, df2)	*P*-value	Group versus ShOPR	Group versus Isch	Group versus Zn+Isch	Group versus IPC+Isch
ShOPR	15	0.84 (0.61)			-	-	-	-
Isch	13	0.59 (0.27)	0.45 (4, 56)		0.207	-	-	-
Zn+Isch	12	0.77 (0.46)		0.770	0.742	0.370	-	-
IPC+Isch	11	0.70 (0.37)			0.503	0.615	0.728	-
Zn+IPC+Isch	12	0.76 (0.69)			0.704	0.397	0.960	0.764

One-way ANOVA, Post-hoc LSD analysis shows no significant difference between

**Table 7:** Comparison of mean serum levels of MDA between the five groups

Groups	*n*	MDA (μmole/L) Mean (SD)	*F*-statistic (df1, df2)	*P*-value	Group versus ShOPR	Group versus Isch	Group versus Zn+Isch	Group versus IPC+Isch
ShOPR	15	9.26 (2.73)			-	-	-	-
Isch	13	11.11 (2.61)	0.76 (4, 56)		0.164	-	-	-
Zn+Isch	12	9.56 (3.91)		0.556	0.821	0.261	-	-
IPC+Isch	11	10.90 (3.37)			0.238	0.879	0.352	-
Zn+IPC+Isch	12	9.71 (4.35)			0.744	0.320	0.918	0.417

One-way ANOVA, Post-hoc LSD analysis shows no significant difference between

**Table 8:** Comparison of mean serum levels of nitrite between the five groups

Groups	*n*	Nitrite (μmole/L) Mean (SD)	*F*-statistic (df1, df2)	*P*-value	Group versus ShOPR	Group versus Isch	Group versus Zn+Isch	Group versus IPC+Isch
ShOPR	15	4.42 (1.79)			-	-	-	-
Isch	13	4.56 (1.86)	0.39 (4, 57)		0.875	-	-	-
Zn+Isch	12	5.05 (1.89)		0.814	0.486	0.593	-	-
IPC+Isch	11	5.35 (3.20)			0.317	0.402	0.755	-
Zn+IPC+Isch	12	5.18 (2.55)			0.401	0.500	0.890	0.859

One-way ANOVA, Post-hoc LSD analysis shows no significant difference between
